# Supplementary material for: Single-nuclei chromatin profiling of ventral midbrain reveals cell identity transcription factors and cell-type-specific gene regulatory variation
Source: Epigenetics Chromatin. 2021 Sep 9;14:43. doi: 10.1186/s13072-021-00418-3 (PMC8427957; doi:10.1186/s13072-021-00418-3)
Supplement: Supplementary file 1 — Additional file 1: Figure S1. snATAC-seq on ventral midbrains of C57BL/6J and A/J revealed cell-type-specific chromatin accessibility. Figure S2. H3K27ac ChIP-seq and ATAC-seq correlating with gene expression. Figure S3. Differential peaks can reveal strain-specific TFs. [file 13072_2021_418_MOESM1_ESM.pdf]

Single nuclei chromatin profiles of ventral midbrain reveal cell identity transcription factors and cell type-specific gene regulatory variation

Yujuan Gui[1], Kamil Grzyb[2], Mélanie H. Thomas[2], Jochen Ohnmacht[1,2], Pierre Garcia[2], Manuel Buttini[2], Alexander Skupin[2], Thomas Sauter[1], Lasse Sinkkonen[1*]

Additional Information

Additional Figures

Figure S1: snATAC-seq on ventral midbrains of C57BL/6J and A/J revealed cell type-specific chromatin accessibility.

A. The ventral midbrains of the two mouse strains were used as input to snATAC-seq. Ten cell types were identified based on clustering of peak features. The aggregated signal is comparable to bulk ATAC-seq. Different accessibility across cell types can be observed and they can reflect gene expression changes. *Prrc2a* is globally accessible and its expression can be detected in all cell types. *Aif1* and *Lst1* TSS are selectively accessible in macrophage, and their expression is only abundant in macrophage.

Figure S2: H3K27ac ChIP-seq and ATAC-seq correlating with gene expression.

A. H3K27ac ChIP-seq on ventral midbrains of C57BL/6J and A/J. Within-sample normalization is applied to account for gene length. The intensity of H3K27ac ChIP-seq signals are plotted in a window of 2000 bp upstream and downstream of gene body. The genes are ordered based on the highest to the lowest gene expression level.

B. ATAC-seq on ventral midbrain of C57BL/6J. The plotting scheme is the same as Supplementary Figure 2A.

Figure S3: Differential peaks can reveal strain-specific TFs.

A. Motif enrichment analysis on differential peaks. The PWM logos, names of the associated TFs and p-values are shown for each motif. The motifs are ranked according to p-values.

Figure S1

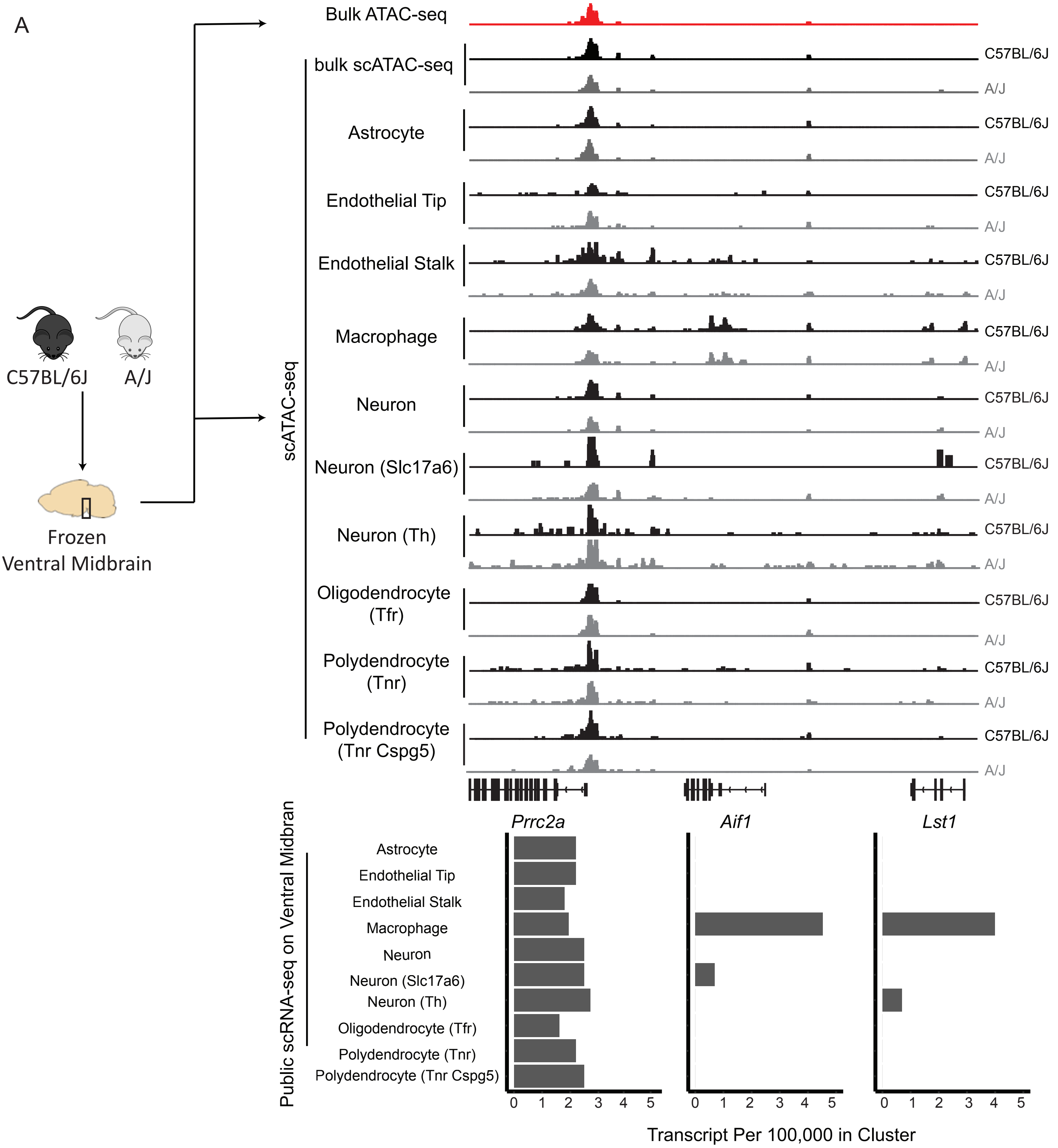
A
31 Kb
Bulk ATAC-seq
bulk scATAC-seq
Astrocyte
Endothelial Tip
Endothelial Stalk
Macrophage
Neuron
Neuron (Slc17a6)
Neuron (Th)
Oligodendrocyte (Tfr)
Polydendrocyte (Tnr)
Polydendrocyte (Tnr Cspg5)
scATAC-seq
C57BL/6J
A/J
Frozen Ventral Midbrain
Prrc2a
Aif1
Lst1
Public scRNA-seq on Ventral Midbran
Astrocyte
Endothelial Tip
Endothelial Stalk
Macrophage
Neuron
Neuron (Slc17a6)
Neuron (Th)
Oligodendrocyte (Tfr)
Polydendrocyte (Tnr)
Polydendrocyte (Tnr Cspg5)
0 1 2 3 4 5
Transcript Per 100,000 in Cluster

Figure S2

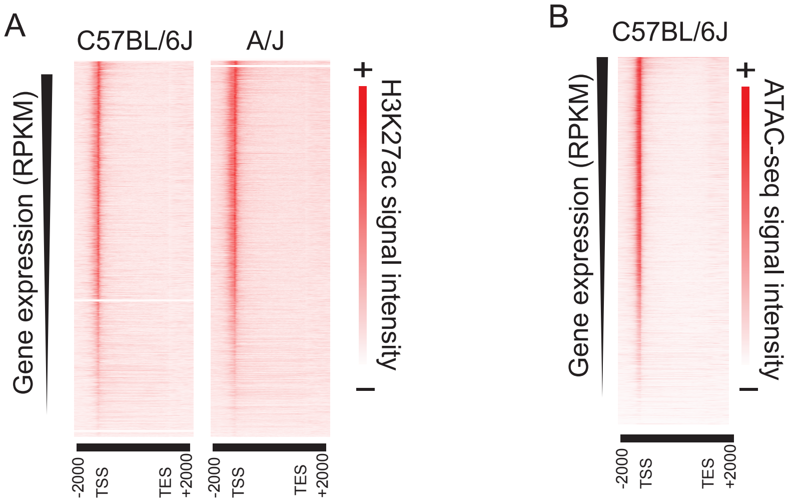
A
C57BL/6J
A/J
Gene expression (RPKM)
H3K27ac signal intensity
+
-
-2000
TSS
TES
+2000
B
C57BL/6J
Gene expression (RPKM)
ATAC-seq signal intensity

Figure S3

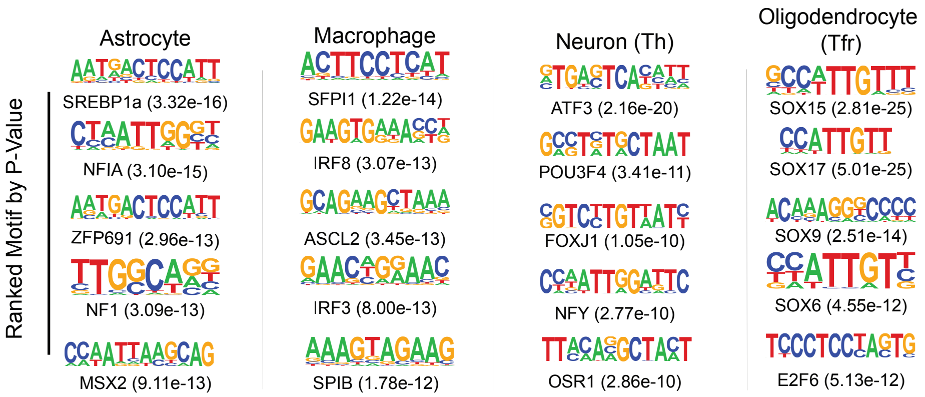
Astrocyte
Macrophage
Neuron (Th)
Oligodendrocyte (Tfr)
Ranked Motif by P-Value
SREBP1a (3.32e-16)
NFIA (3.10e-15)
ZFP691 (2.96e-13)
NF1 (3.09e-13)
MSX2 (9.11e-13)
SFPI1 (1.22e-14)
IRF8 (3.07e-13)
ASCL2 (3.45e-13)
IRF3 (8.00e-13)
SPIB (1.78e-12)
ATF3 (2.16e-20)
POU3F4 (3.41e-11)
FOXJ1 (1.05e-10)
NFY (2.77e-10)
OSR1 (2.86e-10)
SOX15 (2.81e-25)
SOX17 (5.01e-25)
SOX9 (2.51e-14)
SOX6 (4.55e-12)
E2F6 (5.13e-12)

Additional Tables

Table S1: Cell type composition in ventral midbrains of C57BL/6J and A/J in snATAC-seq. Cell types and identified cell numbers are indicated for both strains

Table S2: Cell type-identity genes defined from existing scRNA-seq. The official gene symbols for the identified cell identity genes are listed under each respective cell type.

Table S3: Enrichment analysis on the cell type-identity genes defined from existing scRNA-seq. The GO enrichment results for the cell identity genes for each cell type are provided. The enrichment analysis was performed using Enrichr and GO term identifiers, enrichment p-values, scores, and identified gene names are provided for each GO term.

Table S4: Cell type-identity peaks defined by associating cell type-specific peaks to the regulatory regions of cell type-identity genes. The chromosome coordinates for all cell identity peaks are provided for each cell type.

Table S5: Putative regulatory variants. The chromosome coordinates and major / alternative alleles are reported for the 3909 variants locating within TF footprints in active enhancers and designated as putative regulatory variants.

Table S6: H3K27ac differential peaks between C57BL/6J and A/J. The chromosome coordinates of the peaks and p-values of signal difference between the strains are indicated for each differential peak ($p<1x10^{-18}$).
